# SC-NBTI: A Smart Contract-Based Incentive Mechanism for Federated Knowledge Sharing

**DOI:** 10.3390/s25185802

**Published:** 2025-09-17

**Authors:** Yuanyuan Zhang, Jingwen Liu, Jingpeng Li, Yuchen Huang, Wang Zhong, Yanru Chen, Liangyin Chen

**Affiliations:** 1 College of Computer Science, Sichuan University, Chengdu 610065, China; yuanyuanzhang@stu.scu.edu.cn (Y.Z.); liujw@stu.scu.edu.cn (J.L.); 2021223049281@stu.scu.edu.cn (J.L.); yuchen_huang@stu.scu.edu.cn (Y.H.); zhongwang@stu.scu.edu.cn (W.Z.); chenyanru@scu.edu.cn (Y.C.); 2 Institude for Industrial Internet Research, Sichuan University, Chengdu 610065, China

**Keywords:** incentive mechanism, Nash bargaining, federated learning, smart contract

## Abstract

With the rapid expansion of digital knowledge platforms and intelligent information systems, organizations and communities are producing a vast number of unstructured knowledge data, including annotated corpora, technical diagrams, collaborative whiteboard content, and domain-specific multimedia archives. However, knowledge sharing across institutions is hindered by privacy risks, high communication overhead, and fragmented ownership of data. Federated learning promises to overcome these barriers by enabling collaborative model training without exchanging raw knowledge artifacts, but its success depends on motivating data holders to undertake the additional computational and communication costs. Most existing incentive schemes, which are based on non-cooperative game formulations, neglect unstructured interactions and communication efficiency, thereby limiting their applicability in knowledge-driven scenarios. To address these challenges, we introduce SC-NBTI, a smart contract and Nash bargaining-based incentive framework for federated learning in knowledge collaboration environments. We cast the reward allocation problem as a cooperative game, devise a heuristic algorithm to approximate the NP-hard Nash bargaining solution, and integrate a probabilistic gradient sparsification method to trim communication costs while safeguarding privacy. Experiments on the FMNIST image classification task show that SC-NBTI requires fewer training rounds while achieving 5.89% higher accuracy than the DRL-Incentive baseline.

## 1. Introduction

With the rapid growth of digital platforms and intelligent knowledge systems, researchers and organizations are producing increasing volumes of domain-specific data through collaborative platforms, knowledge graphs, and distributed content creation [1]. However, knowledge sharing in this context faces significant challenges—privacy constraints, lack of proper incentives, and fragmented data ownership across contributors all hinder effective collaboration. Federated learning [2,3] offers great potential by enabling multiple parties to collaboratively train models without sharing raw knowledge assets or proprietary datasets. Yet, most existing research assumes that knowledge holders (e.g., institutions, enterprises, and community networks) will willingly participate. In practice, they face computational burdens, communication overhead, and increased risks related to knowledge leakage and intellectual property, which may discourage their willingness to contribute. Access to diverse, high-quality knowledge is essential to building accurate and fair knowledge-driven systems, underscoring the necessity of robust incentive mechanisms.

Existing research categorizes federated learning incentive mechanisms into three types: reputation-based, payment-based, and game theory-based federated learning [4]. Reputation-based mechanisms [5] reward participants with high historical performance, while payment-based ones [6] directly compensate contributions to encourage data sharing. Both require accurate evaluation to ensure fairness and transparency. Game theory-based mechanisms [7,8] use rewards and penalties to promote rational participation and improve collaboration efficiency. In federated learning [9,10], participant interactions are usually structured and predictable, making non-cooperative game theory widely applicable. However, existing mechanisms often neglect communication efficiency and unstructured interactions, leading to several challenges: first, incentive models [11] based on non-cooperative game theory are primarily suited for structured interactions, but they overlook unstructured interactions, leading to the loss of high-quality data providers; second, incentive mechanisms involving Nash bargaining solutions are typically NP-hard, making them difficult to solve; finally, communication costs in federated learning are often neglected.

To address these challenges, we propose SC-NBTI, an incentive model that integrates smart contracts with Nash bargaining theory. By explicitly modeling the utilities of data requesters and providers, we reformulate the incentive problem as the search for a Nash bargaining solution (NBS), which ensures cooperative and fair surplus allocation in a computationally efficient manner. Compared with reinforcement learning-based methods, SC-NBTI avoids iterative training overhead, and unlike Shapley value-based methods, it scales effectively with the number of participants. To further enhance practicality, we design a client selection strategy that approximates the NBS with significantly reduced complexity, and we employ a probabilistic gradient sparsification method to mitigate communication costs. Experimental results demonstrate that on the FMNIST dataset, SC-NBTI achieves a 5.89% accuracy improvement over the best baseline method, DRL-Incentive, with fewer training rounds.

In summary, the main contributions of this manuscript are as follows:

(1) To address the limitations of previous non-cooperative game-based incentive models, which overlook unstructured interactions and lead to the loss of high-quality data providers, we introduce Nash bargaining theory into the incentive mechanism. We construct a cooperative game model by modeling the participants in federated learning. This reduces the entry barrier to federated learning, attracts higher-quality data, and enables participants to concentrate on improving model performance, fostering mutually beneficial collaboration between data requesters and providers.
(2) To reduce the difficulty of approximating the NP-hard Nash bargaining solution (NBS), we propose a heuristic algorithm that derives an approximate NBS within polynomial time complexity. During the data provider selection phase, we introduce a greedy-based selection strategy and formulate payment strategies based on this selection, significantly improving computational speed and ensuring the efficiency of federated learning.
(3) To address the high communication costs in traditional federated learning, we employ a probabilistic sparsification gradient method, which reduces communication costs while maintaining federated learning quality and partially ensuring data privacy. Experimental results show that SC-NBTI improves model accuracy while ensuring fair allocation. On FMNIST, it achieves a 5.89% accuracy improvement over the best baseline method, DRL-Incentive, with fewer training rounds.

The remainder of this paper is organized as follows: Section 2 examines related work. Section 3 provides a detailed description of the algorithm proposed in this paper. Section 4 analyzes the performance of the algorithm proposed in this paper in different aspects through experiments. Section 5 concludes this paper.

## 2. Related Works

Reputation-based incentive mechanisms [12,13,14] have been widely applied in federated learning [15]. Zhao et al. [5] designed a blockchain-integrated mechanism with dynamic reputation updates to support cooperative training in mobile edge computing, but it is still vulnerable to manipulation, especially with few participants. Kang [16] proposed the TWSL model to manage vehicle reputations through interaction history, enabling high-quality data sharing. While these methods improve data quality and engagement [17], they still face manipulation risks, scalability issues, and complexity in deployment.

Payment-based federated learning incentive mechanisms reward participants based on their contributions to model training. Song et al. [6] introduced a “contribution index” using Shapley values to quantify individual contributions, though its computation is expensive. Wang et al. [18] further developed a scheme for both horizontal and vertical federated learning, using deletion methods for instance-level impact and Shapley values for feature importance. While these methods encourage collaboration, they still face challenges in efficiency and scalability due to the complexity of Shapley value computations.

Game theory-based incentive mechanisms promote participation in federated learning by modeling strategic interactions and payoffs. Zeng et al. [7] proposed a multi-dimensional auction scheme that considers data quality, resources, and network conditions, though inaccurate evaluations may lead to unfair allocation. Lim et al. [8] applied coalition game theory with merge-and-split algorithms to reward marginal contributions, but the approach demands high real-time responsiveness, making it less suitable for unstable networks. Zhan et al. [19] modeled incentives as a Stackelberg game and used deep reinforcement learning to optimize strategies without shared decision data, but performance depends heavily on data quality. While these methods improve engagement and resource use, they face challenges in adaptability and evaluation accuracy.

Blockchain- and smart contract-based incentive mechanisms have recently emerged as an important paradigm to enhance transparency, automation, and trust in federated and distributed learning environments. Zhang et al. [20] proposed a smart contract-based, data-quality-driven incentive mechanism for IoT data sharing which integrates quality evaluation with automated contract execution to ensure high-quality participation under resource constraints. Liu et al. [21] further demonstrated how blockchain-enabled smart contracts can incentivize the dynamic updating and sharing of online learning resources, rewarding both contributors and users in a tamper-proof manner. Yu et al. [22] developed a contract-theoretic incentive framework for resource allocation in MEC-enabled blockchain systems, showing that properly designed contracts can balance miners’ and service providers’ payoffs and maximize social welfare. In the security domain, Wang et al. [23] designed a smart contract-based audit mechanism for DDoS attack traceability in the intelligent IoT, illustrating the potential of blockchain auditing to provide accountability and secure data provenance.

Different from existing cooperative game formulations such as coalition formation and cooperative cost sharing, which usually assume centralized coordination or repeated negotiation, our approach embeds Nash bargaining into smart contracts to realize cooperative surplus sharing in a decentralized and automated manner. Current incentive mechanisms do not adequately consider communication efficiency and unstructured interactions. Additionally, most methods are based on non-cooperative games, making it difficult to handle unstructured interactions, which leads to the loss of high-quality data providers. Furthermore, communication costs in federated learning are often neglected.

## 3. Methodology

### 3.1. Framework of SC-NBTI

**Figure 1:**
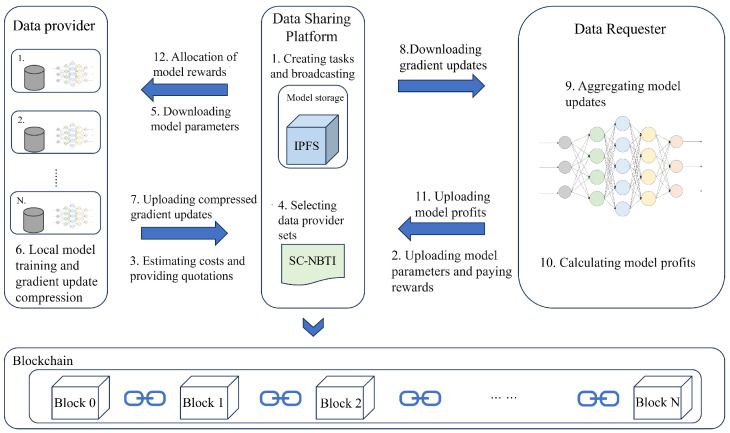
Overall framework of SC-NBTI.

Figure 1 illustrates the overall architecture of SC-NBTI, which enables federated learning between data providers and requesters by leveraging blockchain and the Inter Planetary File System (IPFS). The IPFS serves as a decentralized peer-to-peer storage protocol, ensuring that large artifacts such as model parameters and training metadata are securely maintained off-chain, while their hashes are anchored on-chain for verifiability. To improve communication efficiency, SC-NBTI integrates probabilistic gradient sparsification, which significantly reduces transmission overhead without compromising model accuracy or privacy. A greedy client selection mechanism is employed to approximate the Nash bargaining solution, ensuring fairness and computational tractability in large-scale settings. In addition, a bonus payment strategy is designed to balance participant utilities, incentivizing high-quality contributions and discouraging free-riding. Built upon the Nash Bargaining Theory-based Incentive (NBTI) model and deployed on blockchain [24], SC-NBTI guarantees transparent, automated, and auditable task management. This integration not only strengthens trust among participants but also minimizes disputes and systemic risks, thereby providing a robust foundation for sustainable federated learning ecosystems. The key steps are as follows.

1. The data requester creates a federated learning task and broadcasts the data request information to the relevant community. The information includes the task ID, task budget, required data size, etc.
2. Each data provider uploads the current model to the IPFS, submits the IPFS address to SC-NBTI for management, and pays the model reward for this round.
3. Members who receive the message estimate their computation and communication costs, determine their total cost, and submit a minimum bid along with their local data size to the shared platform.
4. The NBTI smart contract selects a set of data providers using the greedy strategy described in Algorithm 1 of this paper and sends the current IPFS address to the selected data providers.
5. The selected data providers download the model parameters for this round from the IPFS.
6. The data providers train the model using their local data and perform sparsification on the training results as described in Section 3.1 of this paper.
7. The data providers upload the sparsified gradient updates to the IPFS and record the IPFS address on the blockchain.
8. The data requester retrieves the gradient updates from each data provider via the IPFS.
9. The data requester performs model aggregation based on the received gradient updates.
10. The data requester evaluates the model performance for this round.
11. The data requester uploads the model performance (revenue) to NBTI.
12. The NBTI smart contract allocates the reward according to the payment rule in Equation (14), which differentiates the distribution based on model performance.
13. Repeat steps (2) to (12) until the model converges or the specified number of iterations is reached.

**Algorithm 1** Client selection strategy for round *t*.
**Input:**
 

$\left\{K_{m}\right\},\left\{\left|D_{m}\right|\right\},\;B,\;M_{t}$

**Output:**
 Participation Vector for Round *t*: $b_{t}$1:Initialize Parameters: Set all $b_{m}^{t}$ to 02:**if** $M_{t}\leq B$ **then**3:   Set all $b_{t}^{m}$ to 14:
**else**
5:   Calculate $P_{m}^{t}$ using Equation (8)6:   Based on $P_{m}^{t}$, select *B* data providers to form the data provider set *S*7:   Set $b_{m}^{t}$ of each data provider *m* in set *S* to 18:
**end if**
9:

$b_{t}={\left(b_{m}^{t}\right)}_{m\in M_{t}}$

10:**return** $b_{t}$


### 3.2. Communication Optimization Scheme Based on Gradient Sparsification

To reduce communication overhead, we adopt the sparse gradient technique [25], where the local gradient of data provider *m* is compressed into a sparse vector with $K_{m}$ non-zero elements, where $K_{m}<d$ (with *d* being the dimension of the global model). The value of $K_{m}$ is determined by data provider *m* based on its available communication resources. A sparsification probability vector $p\in R^{d}$ is computed, where $p_{i}$ denotes the probability of retaining the *i*-th local gradient element as non-zero and $1-p_{i}$ denotes the probability of dropping the *i*-th element. Gradient elements are randomly dropped according to probability β, and the retained elements are appropriately scaled to ensure that the sparsified gradient remains unbiased. Each provider independently computes a sparsification probability vector $P\in R^{d}$ as in Equation (1), with time complexity O(d).

(1) $$P_{i,m}=\min\left\{\frac{K_{m}\left|g_{m,i}^{t}\right|}{\sum_{i=1}^{d}\left|g_{m,i}^{t}\right|},1\right\}$$

A Bernoulli variable $B_{i}^{m}\in \left\{0,1\right\}$ samples each gradient element, and the sparsification operator $Q:R^{d}\to R^{d}$ is defined in Equation (2). Since $E\left[Q\left(g_{m}^{t}\right)\right]=g_{m}^{t}$, Q(∗) gives an unbiased estimate of $g_{m}^{t}$ with time complexity O(d), enabling efficient gradient sparsification while ensuring convergence.

(2) $$Q\left(g_{m}^{t}\right)={\left[B_{i}^{m}\frac{g_{m,i}^{t}}{P_{i}^{m}}\right]}_{i=1}^{d}$$

### 3.3. Incentive Model Based on Nash Bargaining Theory

Our research considers a federated learning setting with a data requester *R* and data providers *M*. The requester aims to learn a global model $\theta \in R^{d}$, while providers seek rewards.

In the above scenario, we construct a cooperative game model for participants in federated learning based on Nash bargaining theory. Nash bargaining aims to maximize the joint surplus by multiplying each participant’s utility gain relative to their disagreement outcome. In the context of federated learning, this means determining a reward allocation scheme that enables the data requester to train a better global model while ensuring that data providers receive higher rewards. In the following, we model each party involved in federated learning to formulate the corresponding optimization problem and design the incentive mechanism we proposed.

#### 3.3.1. Revenue Modeling for Data Providers

If data provider *m* decides to participate in round *t* of federated learning, then from the perspective of *m*, their utility is the reward $p_{m}^{t}$ obtained from participating in this round. Clearly, $p_{m}^{t}\geq 0$. At the same time, the provider incurs a cost $C_{m}^{t}=C_{m,t}^{\mathrm{cmp}}+C_{m,t}^{\mathrm{com}}$, where $C_{m,t}^{\mathrm{cmp}}$ denotes the computation cost and $C_{m,t}^{\mathrm{com}}$ denotes the communication cost.

We define a binary variable $b_{m}^{t}\in \left\{0,1\right\}$ to indicate whether data provider *m* participates in round *t* of federated learning: if they participate, then $b_{m}^{t}=1$; otherwise, $b_{m}^{t}=0$. For any client *m* in round *t*, the utility can be expressed as the difference between the reward and incurred cost, as shown in Equation (3).

(3) $$V_{m}^{t}\left(b_{m}^{t},p_{m}^{t}\right)=p_{m}^{t}-b_{m}^{t}C_{m}^{t}$$

Here, $b_{m}^{t}$ is the decision made by data provider *m*, while $p_{m}^{t}$ is determined by the data requester *R*. The possible combinations of these decisions by *R* and data provider *m* can be interpreted as whether an agreement is reached for *m* to participate in the federated task in this round. Once decisions are made, the provider’s utility can be computed accordingly.

The total cost of a client can be explicitly modeled as a function of its dataset size $|D_{m}|$ [26]. Specifically, the cost consists of three parts: (i) data cost $c_{m}^{\mathrm{data}}=g_{m}\left|D_{m}\right|$, where $g_{m}$ is the unit data processing cost; (ii) computation cost $c_{m}^{\mathrm{comp}}=a_{m}Md_{l}d_{g}\left|D_{m}\right|$, where *M* is the model dimension, $d_{l}$ and $d_{g}$ are the numbers of local and global iterations, and $a_{m}$ is the unit computation cost; and (iii) communication cost $c_{m}^{\mathrm{comm}}$, which depends on bandwidth, channel gain, and rate constraints but is independent of $|D_{m}|$. Therefore, the total cost is as shown in Equation (4).

(4) $$c_{m}\left(\right|D_{m}\left|\right)=\left(g_{m}+a_{m}Md_{l}d_{g}\right)\;\left|D_{m}\right|+c_{m}^{\mathrm{comm}}$$

This affine form shows that costs grow linearly with dataset size, which plays a key role in ensuring truthfulness in our mechanism.

If data provider *m* decides to participate in the federated learning task for round *t*, they will sparsify their local gradient update according to the method in Section 3.2, upload it to the shared data system, and wait for the aggregated gradient to be used in reward calculation.

#### 3.3.2. Revenue Modeling for Data Requesters

When data requester *R* engages in bargaining with multiple data providers $M_{t}$ during round *t* of a federated learning task, it must first determine which type of bargaining protocol to adopt. Existing one-to-many bargaining protocols include sequential bargaining [27] and parallel bargaining [19]. In sequential bargaining, the requester negotiates with each data provider in a predetermined order, which in the worst case requires time complexity of $O(3^{|M_{t}|})$ [28], making it impractical in real-world data-sharing scenarios.

Therefore, this paper adopts a **parallel bargaining framework for incentive mechanism design. Inspired by the study by Tang [28], we define the utility of the data requester in round *t* as the global model’s accuracy improvement function [29,30,31].

As mentioned above, when data requester *R* receives gradient updates from data providers *m* containing $K_{m}$ non-zero elements, the total number of received sparse gradient parameters $\sum_{m\in M_{t}}b_{m}^{t}K_{m}$ increases, leading to an increase in the model’s overall accuracy $\epsilon \left(\sum_{m\in M_{t}}b_{m}^{t}K_{m}\right)$ [29,30,31].

If no gradient is received (i.e., zero parameters), the model’s accuracy remains ϵ(0). Hence, the accuracy gain during this round of bargaining is defined in Equation (5).

(5) $$f_{t}\left(\sum_{m\in M_{t}}b_{m}^{t}K_{m}\right)=\lambda \left[\varepsilon \left(\sum_{m\in M_{t}}b_{m}^{t}K_{m}\right)-\varepsilon \left(0\right)\right]$$

Here, λ is the amplification coefficient that reflects the data requester’s sensitivity to accuracy improvements.

For data requester *R*, the incurred cost $C_{0}^{t}$ includes both communication cost and payment cost. It is assumed here that the data requester has sufficient communication resources and bears a fixed communication cost $C_{0}^{\mathrm{com}}$ to communicate with the data providers. Therefore, the total communication cost for the data requester is $\sum_{m\in M_{t}}C_{0}^{\mathrm{com}}b_{m}^{t}$. For each data provider *m*, the requester provides a reward $p_{m}^{t}\in R$ for participating in round *t* of federated learning. Obviously, if $b_{m}^{t}=0$, then $p_{m}^{t}=0$, meaning the requester does not pay providers who do not participate.

For simplicity, we define the participation vector $b^{t}≜{\left(b_{m}^{t}\right)}_{m\in M_{t}}$ and the payment vector $p^{t}≜{\left(p_{m}^{t}\right)}_{m\in M_{t}}$. Based on these definitions, the requester’s revenue is given by Equation (6).

(6) $$U\left(b^{t},p^{t}\right)=f_{t}\left(\sum_{m\in M_{t}}b_{m}^{t}K_{m}\right)-\sum_{m\in M_{t}}p_{m}^{t}-\sum_{m\in M_{t}}b_{m}^{t}C_{0}^{com}$$

For any data provider $m\in M_{t}$, if they do not participate in the current round (i.e., $b_{m}^{t}=0$), they will receive no reward (i.e., $p_{m}^{t}=0$) and incur no cost (i.e., $C_{m}^{t}=0$). In this case, their utility is zero (i.e., $V_{m}^{t}\left(0,0\right)=0$). Similarly, if no data provider participates in this round, the data requester’s utility is also evidently zero (i.e., U(0,0)=0). Therefore, the worst-case utility in this bargaining process is 0.

If provider $m\in M_{t}$ does not participate ($b_{m}^{t}=0$), then $p_{m}^{t}=0$, $C_{m}^{t}=0$, and revenue $V_{m}^{t}\left(0,0\right)=0$. If no providers participate, requester revenue U(0,0)=0 is the worst case. After agreeing on $b^{t}$ and $p^{t}$, the requester and providers’ revenues are $U(b^{t},p^{t})$ and $V_{m}^{t}\left(b_{m}^{t},p_{m}^{t}\right)$. By Nash bargaining, the negotiation solves the optimization in Equation (7), equivalently transformed to convex form in Equation (8) via log transform under the same constraints.

(7) $$\begin{array}{c} \max_{b^{t},p^{t}}\;\left[U\left(b^{t},p^{t}\right)-U\left(0,0\right)\right]\prod_{m\in M_{t}}\left[V_{m}^{t}\left(b_{m}^{t},p_{m}^{t}\right)-V_{m}^{t}\left(0,0\right)\right]\\ s.t.\\ V_{m}^{t}\left(b_{m}^{t},p_{m}^{t}\right)-V_{m}^{t}\left(0,0\right)\geq 0,\\ U\left(b^{t},p^{t}\right)-U\left(0,0\right)\geq 0,\\ 1^{T}b^{t}\leq B,\\ 1^{T}b^{t}\leq P_{t},\\ b_{m}^{t}\in \left\{0,1\right\},\;p_{m}^{t}\geq 0,\;p_{m}^{t}\in R,\;\forall m\in M_{t}. \end{array}$$

(8) $$max_{b^{t},p^{t}}\log\left[U\left(b^{t},p^{t}\right)\right]+\sum_{m\in M_{t}}\log\left[V_{m}^{t}\left(b_{m}^{t},p_{m}^{t}\right)\right]$$

The objective in Equation (8) maximizes the joint surplus of the requester and providers above their disagreement points (here U(0,0)=0 and $V_{m}^{t}\left(0,0\right)=0$), which is the classical Nash bargaining criterion (log-sum form after a log transform). In Equation (8), (i) the budget/value constraint ensures that the total payment to providers does not exceed the requester’s available benefit $\min(A,P_{t})$, (ii) the provider cost constraints $p_{m}^{t}\;\geq \;E_{m}^{t}$ guarantee individual rationality (no provider is paid below its incurred cost), and (iii) the non-negativity conditions ensure all parties obtain non-negative utility. Together, these conditions make the outcome both fair and feasible: improving one party’s utility cannot occur at the complete expense of another, and the surplus is split in a way that balances all sides.

Unfortunately, this is a typical Mixed Integer Convex Programming (MICP) problem, which is a classical NP-hard problem. It is difficult to find a globally optimal solution and also challenging to design an algorithm with theoretical approximation guarantees. This difficulty arises because the global utility function and communication costs are determined only after the decisions are made. To address this issue, we propose a heuristic algorithm that derives an approximate Nash bargaining solution within polynomial time complexity. The proposed algorithm is based on the following two key processes: client selection and bonus payment.

#### 3.3.3. Client Selection Strategy

We adopt a non-uniform probabilistic sampling distribution to design the client selection strategy. The proposed strategy is based on the practical observation that the more parameters the data requester receives, the greater the potential utility gain—i.e., the probability that $f_{t}\left(\sum_{m\in M_{t}}b_{m}^{t}K_{m}\right)$ increases is higher [29,30,31]. Since $f_{t}\left(*\right)$ is a non-decreasing function of $K_{m}$, this method assigns a non-zero probability to each data provider *m* based on the size of their local dataset using the Softmax function, thereby enabling the mechanism to better adapt to non-IID data scenarios [32] and ensuring the global model’s convergence [32]. The probability that data provider *m* is selected in the *t*-th round of federated learning is calculated as shown in Equation (9).

(9) $$P_{m}^{t}=\frac{\exp\left(K_{m}+\left|D_{m}\right|-C_{m}^{t-1}\right)}{\sum_{n\in M_{t}}\exp\left(K_{n}+\left|D_{n}\right|-C_{n}^{t-1}\right)}$$

As provider *m* increases reported cost $C_{m}^{t-1}$, their selection probability decreases, while underbidding lowers rewards, incentivizing truthful reporting. Since costs correlate with dataset size $\left|D_{m}\right|$ [26], misreporting data size has similar effects, encouraging truthfulness.

Although Equation (9) suggests that a client might increase its reported $|D_{m}|$ to gain a higher selection probability, the affine cost function above guarantees that such misreporting cannot improve net utility. If a client is already selected truthfully, exaggerating $|D_{m}|$ does not change the allocation or payment but increases the incurred cost $c_{m}\left(\right|D_{m}\left|\right)$, reducing utility. If a client is not selected truthfully, inflating $|D_{m}|$ may cross the selection threshold, but the payment is determined by the critical type (threshold), not by the inflated report. Since the cost strictly increases with $|D_{m}|$, the utility under misreporting cannot exceed that under truthful reporting. Therefore, under monotone allocation and threshold-based payments, truth telling is a dominant strategy, and our client selection strategy satisfies the truthfulness property.

Based on this, we design a probabilistic client selection: At round *t*, set all $b_{m}^{t}=0$. If providers ≤B, select all; else, compute selection probabilities via Equation (9), and randomly sample *B* clients to form set *S*. Return participation vector $b_{t}$. Algorithm 1 runs in $O(M_{t})$ time.

#### 3.3.4. Bonus Payment Strategy

For simplicity, we define $A=f_{t}\left(\sum_{m\in M_{t}}b_{m}^{t}K_{m}\right)-\sum_{m\in M_{t}}b_{m}^{t}C_{0}^{m}$ as the data requester’s net benefit in round *t* and $E_{m}^{t}=b_{m}^{t}C_{m}^{t}$ as provider *m*’s incurred cost. Substituting these into Equation (8), we derive the equivalent optimization in Equation (10) and, by reorganizing the constraints, express the problem equivalently as Equation (11).

(10) $$\begin{array}{cc} \; & max_{p^{t}}\log\left(A-\sum_{m\in S_{t}}p_{m}^{t}\right)+\sum_{m\in S_{t}}\log\left(p_{m}^{t}-E_{m}^{t}\right)\\ \; & \;\;\;s.t.\left\{\begin{array}{c} p_{m}^{t}-E_{m}^{t}\geq 0\\ A-\sum_{m\in S_{t}}p_{m}^{t}\geq 0\\ \sum_{m\in S_{t}}p_{m}^{t}\leq P_{t}\\ p_{m}^{t}\geq 0,p_{m}^{t}\in R,\forall m\in S_{t} \end{array}\right. \end{array}$$

(11) $$\begin{array}{cc} \; & min_{p^{t}}-\log\left(A-\sum_{m\in S_{t}}p_{m}^{t}\right)-\sum_{m\in S_{t}}\log\left(p_{m}^{t}-E_{m}^{t}\right)\\ \; & \;\;\;s.t.\left\{\begin{array}{c} \sum_{m\in S_{t}}p_{m}^{t}\leq min\left(A,P_{t}\right)\\ p_{m}^{t}-E_{m}^{t}\geq 0\\ p_{m}^{t}\in R,\forall m\in S_{t} \end{array}\right. \end{array}$$

Given the convex nature of Equation (11), we apply the Karush–Kuhn–Tucker (KKT) conditions to characterize its optimal solution. By introducing Lagrange multipliers θ and $V=[v_{1},\ldots ,v_{m},\ldots ,v_{M_{t}}]\geq 0$ to constrain $\sum_{m\in S_{t}}p_{m}^{t}\leq \min\left(A,P_{t}\right)$ and $P_{m}^{t}\geq E_{m}^{t}$, we derive the KKT conditions as in Equation (12). Solving them yields the closed-form solution in Equation (13), from which the optimal payment $p_{m}^{t}$ for selected providers can be computed (Equation (14)).

From these conditions, the complementary slackness relations imply that the optimal solution must satisfy $p_{m}^{t}\geq E_{m}^{t}$ for each selected provider and that the total payment cannot exceed the requester’s budget or task value. Rearranging the first-order condition in Equation (12) yields a system of linear equations in the payment variables, where each $p_{m}^{t}$ depends on the requester’s budget *A*, minimum cost $E_{m}^{t}$, and the payments of other selected providers. Solving this system leads to the recursive form shown in Equation (13).

Finally, under the mild symmetry assumption that all selected providers are treated homogeneously in equilibrium, the surplus can be evenly divided among the $|S_{t}|$ providers plus the requester. This simplification yields the closed-form expression in Equation (14), where each selected provider receives a payment proportional to its minimum cost and the requester’s budget. This step makes explicit the assumption of symmetric equilibrium and explains the transition from Equation (13) to Equation (14).

(12) $$\left\{\begin{array}{c} \frac{1}{A-\sum_{m\in M_{t}}p_{m}^{t}}-\frac{1}{p_{m}^{t}-E_{m}^{t}}+\theta -v_{m}=0,\forall m\in M_{t}\\ \sum_{m\in M_{t}}p_{m}^{t}\leq min\left(A,P_{t}\right)\\ \theta \geq 0\\ \theta \left(\min\left(A,P_{t}\right)-\sum_{m\in M_{t}}p_{m}^{t}\right)=0\\ p_{m}^{t}-E_{m}^{t}\geq 0,\forall m\in M_{t}\\ v_{m}\left(p_{m}^{t}-E_{m}^{t}\right)=0,\forall m\in M_{t}\\ v_{m}\geq 0 \end{array}\right.$$

(13) $${\tilde{p}}_{m}^{t}=\frac{A+E_{m}^{t}-\sum_{n\in S_{t}\setminus \left\{m\right\}}{\tilde{p}}_{n}^{t}}{2},\forall m\in S_{t}$$

(14) $${\tilde{p}}_{m}^{t}=\frac{A+E_{m}^{t}}{\left|S_{t}\right|+1}$$

Equation (14) distributes the cooperative surplus between the requester and the $|S_{t}|$ selected providers in a Nash-consistent manner: each provider’s payment increases with its minimum cost $E_{m}^{t}$ (ensuring individual rationality), while the requester’s share is implicitly reflected through the denominator $|S_{t}|\;+\;1$. This is a differentiated allocation (not an equal split) in general; it reduces to equal sharing only under symmetry.

The overall social welfare generated by federated learning is shared between the requester and the providers, ensuring aligned interests and encouraging cooperation. As in Section 3.3.3, our incentive mechanism prevents cost or data misreporting and stops the requester from undervaluing benefits to cut costs. Providers can verify accuracy gains locally each round, ensuring transparency. As discussed in Section 3.4, smart contracts provide an additional layer of assurance. The full process is detailed in Algorithm 2.
**Algorithm 2** Incentive mechanism.**Input:**
 $T,\;\lambda ,\;B,\;\eta ,\;M_{t},\;\left\{K_{m}\right\},\;\{|D_{m}\left|\right\}$**Output:**
 Global model $\theta_{T}$1:Initialization: global model $\theta_{0}$, initial accuracy ϵ(0)2:**for**  t=1 to *T* **do**3:   $b_{t}\leftarrow \mathrm{Algorithm 1}\;\;\;\;\left(M_{t},\;B,\;\left\{K_{m}\right\},\;\{|D_{m}\left|\right\}\right)$4:   Select data providers $S_{t}$ for round *t* based on budget $b_{t}$5:   Send the latest global model $\theta_{t-1}$ to each data provider in $S_{t}$6:   Each data provider $m\;\in \;S_{t}$ trains a local model and obtains local gradient $g_{m}^{t}$7:   Each data provider $m\;\in \;S_{t}$ sparsifies $g_{m}^{t}$ using Equation (2) and uploads it8:   Parameter aggregation:$$\Delta_{t}\;=\;\frac{1}{|S_{t}|}\sum_{m\in S_{t}}\frac{Q\;\left(g_{m}^{t}\right)}{P_{m}^{t}},\;\theta_{t}\;=\;\theta_{t-1}-\eta \;\Delta_{t}$$9:   Compute:$$A\;=\;f_{t}\;\left(\sum_{m\in S_{t}}b_{m}^{t}K_{m}\right)\;-\;\sum_{m\in S_{t}}b_{m}^{t}C_{0}^{m},\;E_{m}^{t}\;=\;b_{m}^{t}C_{m}^{t},\;\;\forall m\in S_{t}$$10:   Compute the payment amount for each data provider in the current round according to Equation (14)11:**end for**12:**return** $\theta_{T}$

### 3.4. Smart Contract Design Based on NBTI

**Figure 2:**
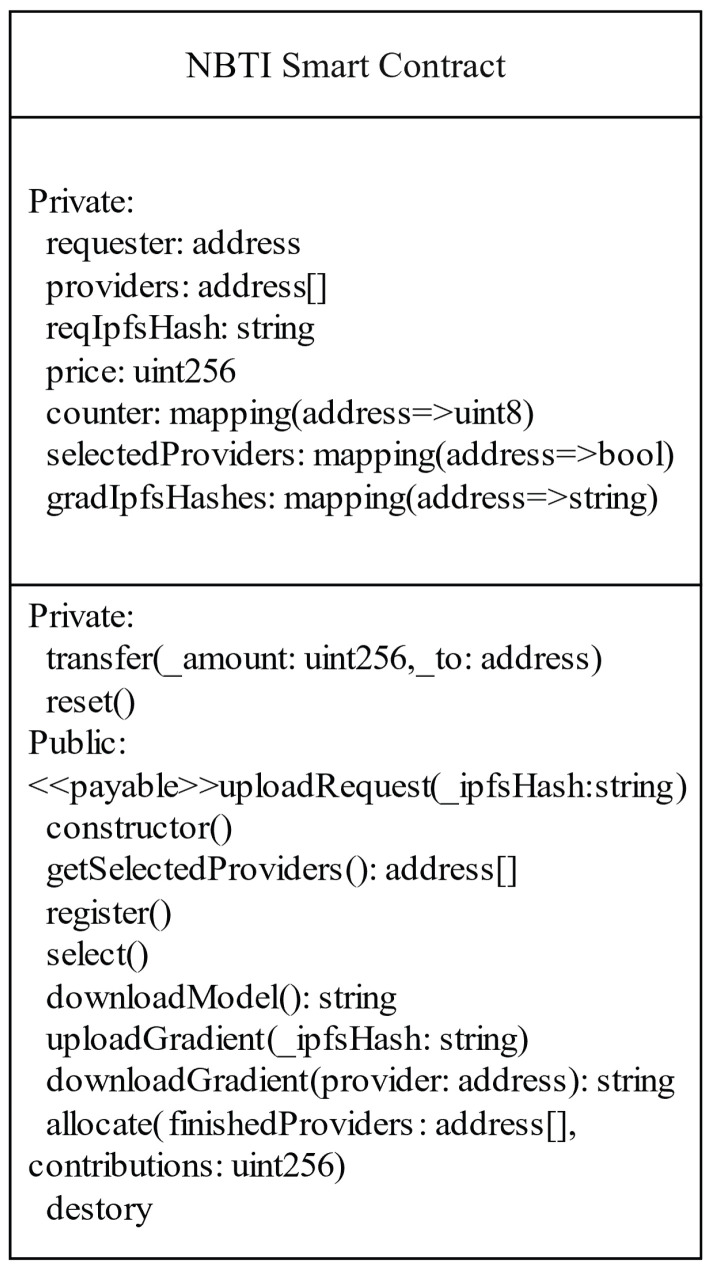
The UML diagram of NBTI.

In NBTI, the requester initiates and supervises the federated learning task, while cooperative game theory reduces strategic deception and ensures fair benefit allocation. Smart contracts enforce compliance, detect potential violations, and guarantee transparency, fairness, and immutability, thereby enhancing system reliability. The SC-NBTI model is implemented via blockchain smart contracts, as depicted in the UML diagram in Figure 2.

The contract encapsulates key private variables, such as the requester, the list of providers, task price, model and gradient IPFS addresses, and mappings for provider selection and contribution tracking. By storing only IPFS addresses on-chain, the design minimizes storage costs and leverages decentralized off-chain storage for large artifacts. In addition to the constructor and destructor, the contract provides eight major public functions that govern the lifecycle of one federated learning round:

1. uploadRequest(): It allows the requester to upload the IPFS address of the initial model parameters and deposit the corresponding reward. This ensures task transparency and secures the requester’s commitment.
2. getSelectedProviders(): It returns the addresses of providers selected for the current round, enabling verifiable participation.
3. register(): It provides a registration interface for candidate providers, ensuring that only authenticated participants can join.
4. select(): It implements the greedy client selection mechanism described in this paper, approximating the Nash bargaining solution while maintaining computational efficiency.
5. downloadModel(): It grants selected providers access to the model via the IPFS, ensuring decentralized and auditable distribution.
6. uploadGradient(): It enables providers to submit sparsified gradient updates through the IPFS, which are then referenced on-chain for verifiability.
7. downloadGradient(): It allows the requester to retrieve submitted gradient updates for model aggregation.
8. allocate(): It executes the allocation of rewards to providers based on their contributions, closing the incentive loop and updating reputational records.

The meanings of other related variables are shown in Table 1.

## 4. Experimental Analysis and Discussion

### 4.1. Performance Comparison Experiment

#### 4.1.1. Experimental Setup

We conducted experiments using the Plato federated learning framework based on PyTorch (2.2.1). Different threads were used to simulate various clients. Additionally, we employed the Dirichlet distribution to model non-IID (non-independent and identically distributed) data scenarios. The Dirichlet distribution parameters were set to 0.1 to simulate heterogeneous environments.

We used three classic computer vision datasets in our experiment, CIFAR-10 [33], SVHN [34], and FMNIST [35]:

(1) CIFAR-10: It contains 60,000 32 × 32 color images across 10 classes (e.g., airplanes, cars, cats, etc.), widely used for image classification benchmarks.
(2) SVHN: A dataset with over 600,000 color images of digits (0–9) from Google Street View, ideal for studying digit recognition in real-world scenarios.
(3) FMNIST: It includes 70,000 28 × 28 grayscale images of clothing items in 10 categories, often used as a more complex alternative to MNIST for testing algorithms.

**Figure 3:**
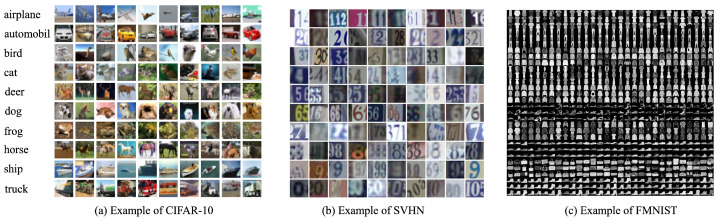
Example of three datasets.

Dataset examples are shown in Figure 3. The objective of the experiment is to train a moderately sized Convolutional Neural Network (CNN) as the global model. In each round, the number of non-zero elements in the sparse gradient vector of client *m*, denoted by $K_{m}$, is randomly selected from the set 1000, 2000, 3000, 4000, 5000, 6000, 7000, 8000, 9000, 10,000. The experiment simulates a federated learning interaction between 1 server (i.e., the data requester) and 100 threads (i.e., data providers). The client cost and data size are configured using a uniform distribution over the interval [50,100]. In each round, 20 clients are activated to participate in the bargaining process. For simplicity, the following hyperparameters are used: the learning rate is set to η=0.1, the gain coefficient is set to λ=5000, and the batch size for data loading is set to 32.

We select four commonly used federated learning incentive mechanisms as baselines for comparison: DRL-Incentive (incentive mechanism based on deep reinforcement learning) [36], Auction-Incentive (incentive mechanism based on auction) [7], Contract-Incentive (incentive mechanism based on contract theory) [37], and Stackelberg-Incentive (incentive mechanism based on Stackelberg game) [38]. We compare their performance on different datasets in terms of the changes in model accuracy and training loss over training rounds, as well as the number of training rounds required to reach a target accuracy.

#### 4.1.2. Experimental Results and Analysis

Each experiment was repeated 10 times, and the average results are reported to reduce random errors and enhance statistical robustness. Repeating the experiments ensures statistical significance, while averaging the results provides a more accurate assessment of model consistency and generalization under different data distributions. The consistent results across multiple runs reinforce the validity of our conclusions.

**Figure 4:**
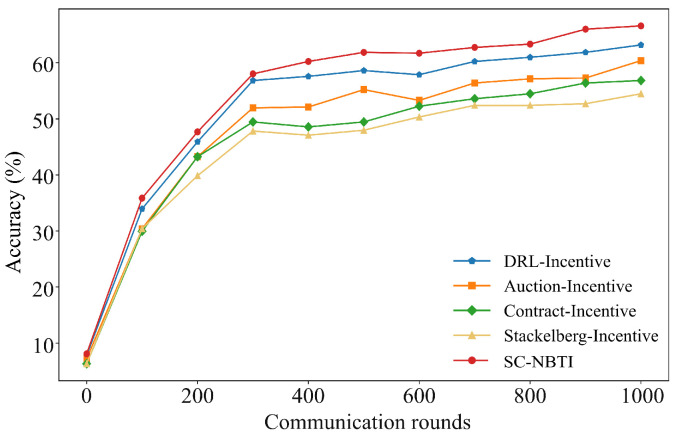
Comparison of Top-1 accuracy of each scheme on CIFAR-10.

**Figure 5:**
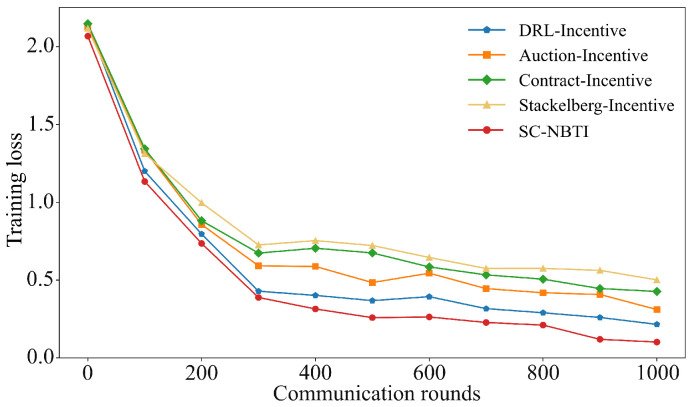
Comparison of training loss of each scheme on CIFAR-10.

Figure 4 and Figure 5 compare the performance of the four baselines and our method on CIFAR-10. All methods show reduced training loss and improved accuracy with more communication rounds, eventually converging to stable levels. Among the baselines, DRL-Incentive achieves the highest accuracy (63.2%), followed by Auction-Incentive (60.39%) and Contract-Incentive (56.60%). Our method outperforms the best baseline by 2.4%, demonstrating superior effectiveness in incentivized federated learning.

Table 2 compares the rounds required for the four baseline methods and SC-NBTI to achieve 60% model accuracy on the CIFAR-10 dataset.

As shown in Table 2, in the CIFAR-10 experiments, our method required only 399 training rounds to achieve 60% accuracy, while DRL-Incentive requires 680 rounds, Auction-Incentive requires 979 rounds, and Contract-Incentive and Stackelberg-Incentive fail to reach 60% accuracy within 1000 rounds. Notably, our method achieves the target accuracy 281 rounds earlier than the best-performing baseline.

There may be two main reasons for this:

(1) The impact of non-IID data on the convergence process of federated learning. Compared with the baselines, our incentive mechanism considers probabilistic client selection, which alleviates the non-IID issue to some extent, thereby reducing the number of communication rounds required to train the global model.
(2) Other baselines are unable to optimize payments to incentivize high-quality data providers. In fact, DRL-Incentive, Auction-Incentive, and Contract-Incentive only ensure non-negative utility for data providers but cannot effectively optimize their rewards through payment schemes. As a result, they fail to fully incentivize high-quality users with more communication resources to participate in the federated learning task.

**Figure 6:**
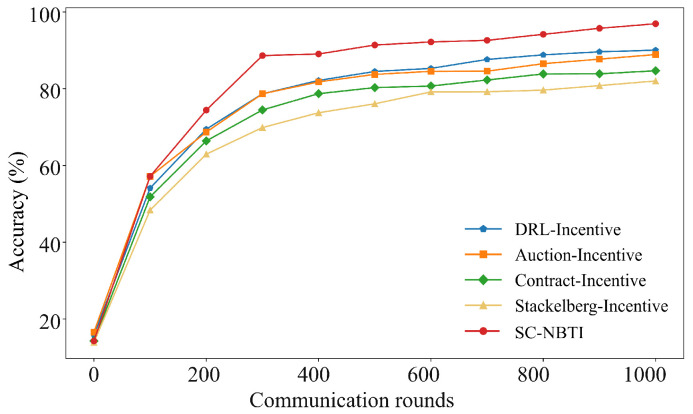
Comparison of Top-1 accuracy of each scheme on FMNIST.

**Figure 7:**
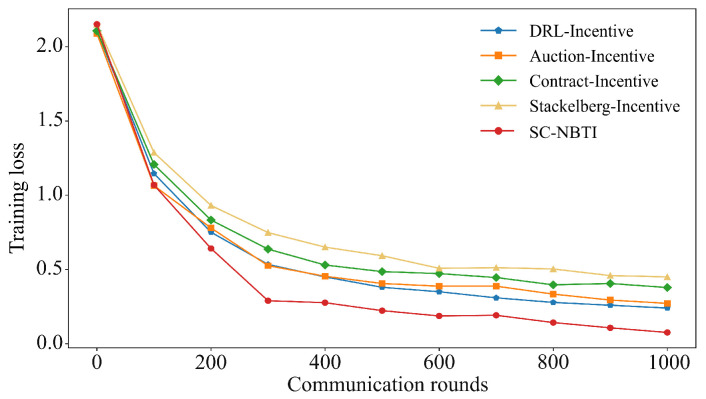
Comparison of training loss of each scheme on FMNIST.

Figure 6 and Figure 7 present the results on the FMNIST dataset. Among the baselines, DRL-Incentive achieves the highest accuracy (90.07%), followed by Auction-Incentive (88.92%), Contract-Incentive (84.71%), and Stackelberg-Incentive (82.03%). Our method reaches 95.96%, surpassing the best baseline by 5.89% and confirming its superior performance in federated learning with incentives.

As shown in Table 3, under our experimental settings on the FMNIST dataset, our method requires only 240 training rounds to reach 80% model accuracy, while DRL-Incentive requires 324 rounds, Auction-Incentive requires 328 rounds, Contract-Incentive requires 477 rounds, and Stackelberg-Incentive requires 812 rounds. Our method reaches the target accuracy 84 rounds earlier than the best-performing baseline.

**Figure 8:**
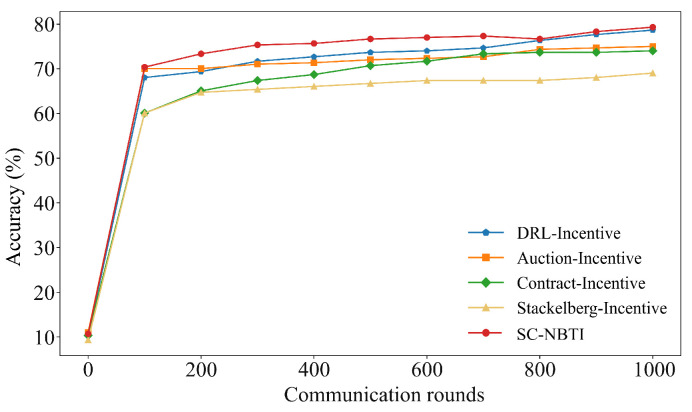
Comparison of Top-1 accuracy of each scheme on SVHN.

**Figure 9:**
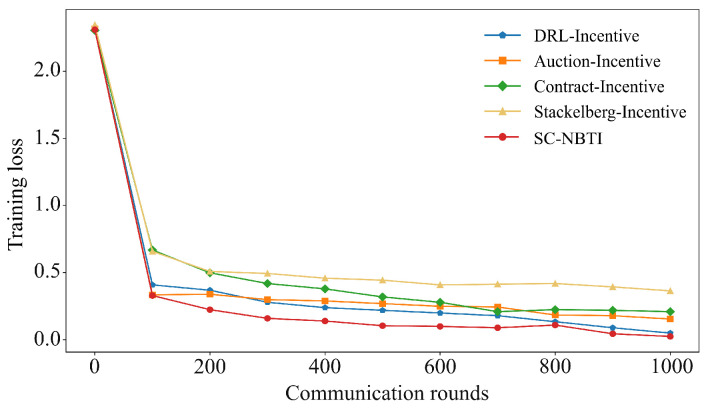
Comparison of training loss of each scheme on SVHN.

Figure 8 and Figure 9 report the results on the SVHN dataset. DRL-Incentive achieves the highest baseline accuracy (78.67%), followed by Auction-Incentive (75.02%), Contract-Incentive (74.03%), and Stackelberg-Incentive (69.05%). Our method attains 79.34%, exceeding the best baseline by 0.67% and demonstrating consistent improvements across datasets.

As shown in Table 4, under our experimental settings on the SVHN dataset, our method requires only 272 training rounds to reach 75% model accuracy, while DRL-Incentive requires 707 rounds, Auction-Incentive requires 977 rounds, and Contract-Incentive and Stackelberg-Incentive fail to reach 75% model accuracy within 1000 rounds. Our method reaches the target accuracy 435 rounds earlier than the best-performing baseline. This is attributed to the fact that our method can quickly incentivize more users to participate in the federated learning task.

### 4.2. Fairness Comparison Experiment

#### 4.2.1. Experimental Setup

**Figure 10:**
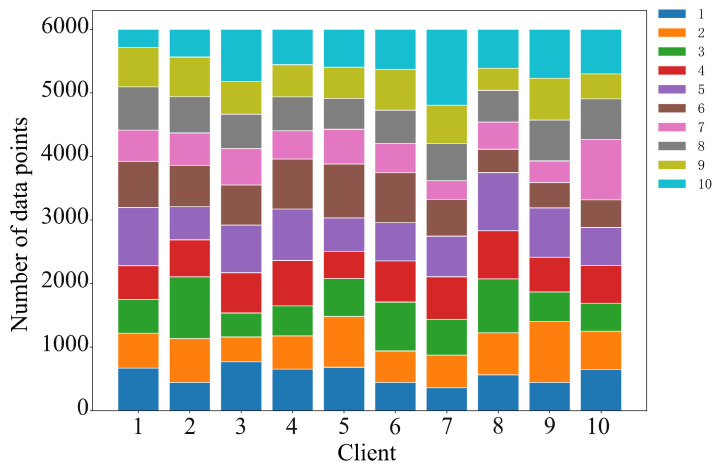
Uniform data distribution diagram.

**Figure 11:**
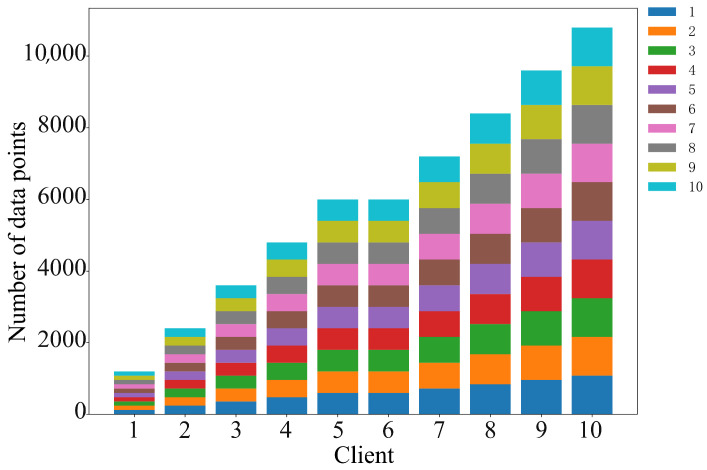
Imbalanced data distribution diagram.

To evaluate the fairness of the SC-NBTI incentive mechanism, we design two different data scenarios based on the MNIST dataset for testing. The data provider distributions corresponding to the two scenarios are shown in Figure 10 and Figure 11.

(1) Random uniform allocation: The MNIST dataset is randomly divided into 10 equal parts and assigned to each data provider.
(2) Uneven data volume: The MNIST dataset is split and assigned to data providers according to the overall data proportions of 2%, 4%, 6%, 8%, 10%, 10%, 12%, 14%, 16%, and 18%.

This section is only intended to compare the allocation fairness in different scenarios, so the client selection process is simplified by assuming that all 10 clients participate in the federated learning task without any bargaining. Meanwhile, the reward per round is set to 2, and the number of federated learning rounds is set to 50.

#### 4.2.2. Experimental Results and Analysis

**Figure 12:**
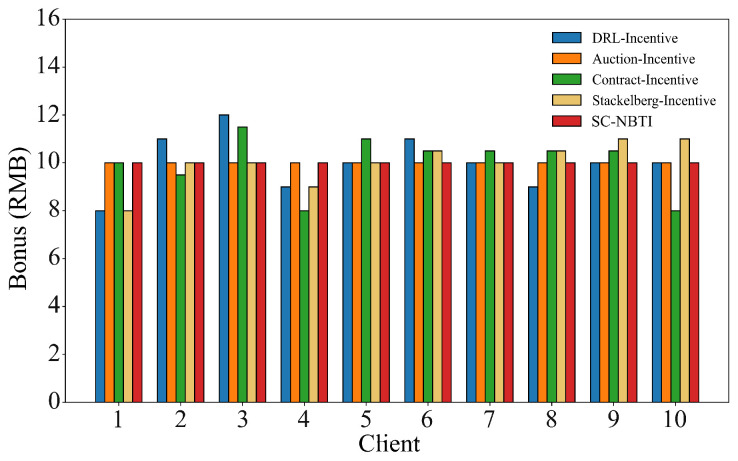
Rewards for each scheme with random uniform data distribution.

**Figure 13:**
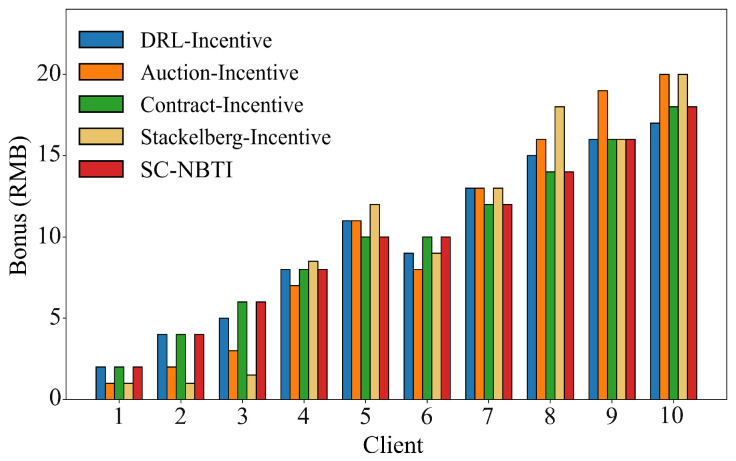
The rewards of each scheme when the number of data is unbalanced.

Figure 12 and Figure 13 show the bonus allocation results of each incentive mechanism under the random uniform partitioning and uneven data volume scenarios, respectively. All methods can correctly evaluate the data quality of data providers and complete the bonus allocation according to their rules. Our method provides a payment scheme in both data scenarios that satisfies the payment strategy defined in Equation (14), which aligns with our expectations.

### 4.3. System Overhead Analysis

In addition to accuracy and training rounds, it is important to consider system-level performance metrics. First, the computational overhead of probabilistic gradient sparsification is relatively lightweight, since the sparsification operator Q(·) has time complexity O(d), where *d* is the model dimension. This ensures that the additional computation does not outweigh the communication savings. Second, smart contract execution may introduce latency due to transaction confirmation on the blockchain; however, only lightweight operations such as storing IPFS addresses and reward allocations are executed on-chain, while heavy computations remain off-chain, thus mitigating overhead. Finally, resource consumption primarily arises from IPFS storage and blockchain transactions. Redundant storage across IPFS nodes provides robustness at the cost of additional disk usage, and blockchain interactions incur gas fees but remain bounded as the number of on-chain operations per training round is constant.

### 4.4. Scalability and Deployment Feasibility

Although the proposed SC-NBTI framework ensures transparency and fairness through blockchain and smart contracts, it also introduces certain scalability concerns. First, smart contract execution may cause additional latency and incur gas costs, which could affect the responsiveness of task allocation and settlement in large-scale deployments. To mitigate this issue, only lightweight information such as IPFS addresses and cryptographic hashes are stored on-chain, while computationally intensive operations are performed off-chain. Second, the use of the IPFS as decentralized storage may lead to reliability and retrieval delay concerns. This can be alleviated by replicating files across multiple IPFS nodes and adopting caching strategies to improve data availability.

### 4.5. Privacy Preservation and Leakage Risks

Although SC-NBTI conceptually incorporates privacy protection, it is important to assess potential leakage risks in practice. In the proposed design, privacy is mainly preserved through two mechanisms: (i) probabilistic gradient sparsification, which reduces the dimensionality of transmitted updates and makes it more difficult for adversaries to infer raw data, and (ii)on-chain/off-chain separation, where sensitive model parameters and gradient values are stored in the IPFS rather than directly on-chain, thereby avoiding unnecessary exposure. Nevertheless, model updates may still contain statistical patterns that could be exploited for data reconstruction or membership inference. To address this, we provide a qualitative discussion of the leakage risks during gradient exchange and highlight that stronger cryptographic techniques such as differential privacy or secure aggregation could be integrated into SC-NBTI to further mitigate these risks. Future work will focus on combining these methods with the current incentive mechanism to achieve a stronger balance among utility, efficiency, and privacy.

## 5. Conclusions

Modern digital collaboration platforms produce increasingly large volumes of unstructured knowledge data, ranging from annotated documents and research corpora to shared whiteboard content and domain-specific media archives. Yet sharing these knowledge assets across institutions, communities, and contributors is hindered by strict privacy regulations, high communication overhead, and misaligned incentives. To tackle these challenges, we presented SC-NBTI: a cooperative incentive framework that embeds Nash bargaining-based reward allocation in tamper-proof smart contracts, augments federated training with probabilistic gradient sparsification to cut communication costs, and uses a lightweight heuristic to approximate the NP-hard bargaining solution. Our experiments on the FMNIST benchmark—as a stand-in for collaborative knowledge classification tasks—demonstrated that SC-NBTI not only reduced the number of training rounds but also achieved a 5.89% accuracy gain over the DRL-Incentive baseline. Although the proposed SC-NBTI framework demonstrates the feasibility of integrating Nash bargaining-based incentives with federated learning, several limitations should be acknowledged. First, the probabilistic gradient sparsification strategy, while effective in reducing communication overhead, may lead to degraded performance in highly non-IID scenarios, as it can amplify divergence among heterogeneous client updates and slow convergence. Second, the current formulation assumes symmetry among selected providers and models communication cost in a simplified affine form, which may not fully capture the complexity of real-world heterogeneous environments. Finally, while the framework has been conceptually validated, domain-specific evaluation and deployment feasibility in sectors such as healthcare or the Internet of Things (IoT) remain unexplored. Future work will, therefore, focus on three directions: (i) developing adaptive sparsification strategies, importance-aware gradient selection, and hybrid approaches that combine sparsification with client clustering or personalized models to better cope with non-IID data distributions; (ii) refining the theoretical formulation by introducing domain-specific contribution metrics and more realistic cost models that reflect heterogeneity in communication and computation; (iii) extending the empirical validation of SC-NBTI to real-world domains such as healthcare and the IoT and exploring hybrid federated optimization schemes that balance efficiency, robustness, and privacy in practical deployment scenarios.

## Figures and Tables

**Table 1:** The meaning of smart contract-related variables based on the NBTI model.

Variable Name	Meaning
requester	Address of the data requester
provider	Address of the data provider
reqIpfsHash	IPFS hash of the model uploaded by the requester
price	Federated learning task budget
counter	Total number of data providers
selectedProviders	Data providers selected for the federated task
bids	Bids submitted by data providers
gradIpfsHashes	IPFS hashes of the gradients from data providers

**Table 2:** Comparison of training rounds to reach 60% accuracy on CIFAR-10.

Method	Number of Rounds
DRL-Incentive	680
Auction-Incentive	979
Contract-Incentive	Did not meet 60%
Stackelberg-Incentive	Did not meet 60%
SC-NBTI	399

**Table 3:** Comparison of training rounds to reach 80% accuracy on FMNIST.

Method	Number of Rounds
DRL-Incentive	324
Auction-Incentive	328
Contract-Incentive	477
Stackelberg-Incentive	812
SC-NBTI	240

**Table 4:** Comparison of training rounds to reach 75% accuracy on SVHN.

Method	Number of Rounds
DRL-Incentive	707
Auction-Incentive	977
Contract-Incentive	Did not meet 75%
Stackelberg-Incentive	Did not meet 75%
SC-NBTI	272

## Data Availability

The data used to support the findings of this study are available from the corresponding author upon request.

## List of Symbols

To improve readability, we summarize the main mathematical symbols used throughout this paper. This provides readers with a quick reference to the meaning of each notation, especially when following the technical details in the Methodology and Analysis Sections.

List of mathematical symbols and their meanings.	
**Symbol**	**Meaning**
*R*	Data requester in federated learning
*M*	Set of data providers
$\theta \in R^{d}$	Global model parameters with dimension *d*
$b_{m}^{t}\in \left\{0,1\right\}$	Participation indicator of provider *m* in round *t*
$p_{m}^{t}\geq 0$	Payment (reward) allocated to provider *m* in round *t*
$C_{m}^{t}$	Total cost of provider *m* in round *t*
$C_{m,t}^{cmp}$	Computation cost of provider *m* in round *t*
$C_{m,t}^{com}$	Communication cost of provider *m* in round *t*
$|D_{m}|$	Local dataset size of provider *m*
$g_{m}$	Unit data processing cost of provider *m*
$a_{m}$	Unit computation cost of provider *m*
*M* (model dim.)	Dimension of the global model
dl,dg	Number of local iterations and global iterations
$K_{m}$	Number of non-zero elements in the sparse gradient of provider *m*
ϵ(·)	Model accuracy function
λ	Amplification coefficient of accuracy improvement
$U(b^{t},p^{t})$	Utility of the requester in round *t*
$V_{m}^{t}\left(b_{m}^{t},p_{m}^{t}\right)$	Utility of provider *m* in round *t*
*A*	Net benefit of the requester in round *t*
$E_{m}^{t}$	Incurred cost of provider *m* in round *t*
$P_{m}^{t}$	Probability of selecting provider *m* in round *t*
Q(·)	Gradient sparsification operator
$g_{m}^{t}$	Local gradient of provider *m* in round *t*
$B_{m}^{i}$	Bernoulli sampling variable for sparsification
$Q(g_{m}^{t})$	Sparsified gradient vector of provider *m*
$S_{t}$	Selected provider set in round *t*
*T*	Total number of communication rounds
η	Learning rate for model update

## References

[1] Chrysafiadi K., Papadimitriou S., Virvou M. (2022). Cognitive-based adaptive scenarios in educational games using fuzzy reasoning. Knowl.-Based Syst..

[2] Chen C., Pan H., Zhang K., Li Z., Yu F. (2025). Prototype-based Personalized Federated Learning for medical image classification. Knowl.-Based Syst..

[3] Zhao H., Sui D., Wang Y., Ma L., Wang L. (2025). Privacy-Preserving Federated Learning Framework for Multi-Source Electronic Health Records Prognosis Prediction. Sensors.

[4] Yang Y., Hu M., Zhou Y., Liu X., Wu D. (2023). Csra: Robust incentive mechanism design for differentially private federated learning. IEEE Trans. Inf. Forensics Secur..

[5] Zhao Y., Zhao J., Jiang L., Tan R., Niyato D. (2019). Mobile edge computing, blockchain and reputation-based crowdsourcing iot federated learning: A secure, decentralized and privacy-preserving system. arXiv.

[6] Song T., Tong Y., Wei S. (2019). Profit allocation for federated learning. Proceedings of the 2019 IEEE International Conference on Big Data (Big Data).

[7] Zeng R., Zhang S., Wang J., Chu X. (2020). FMore: An incentive scheme of multi-dimensional auction for federated learning in MEC. Proceedings of the 2020 IEEE 40th international conference on distributed computing systems (ICDCS).

[8] Lim W.Y.B., Xiong Z., Miao C., Niyato D., Yang Q., Leung C., Poor H.V. (2020). Hierarchical incentive mechanism design for federated machine learning in mobile networks. IEEE Internet Things J..

[9] Li H., Cai Z., Wang J., Tang J., Ding W., Lin C.T., Shi Y. (2023). Fedtp: Federated learning by transformer personalization. IEEE Trans. Neural Netw. Learn. Syst..

[10] Sattler F., Wiedemann S., Müller K.R., Samek W. (2019). Robust and communication-efficient federated learning from non-iid data. IEEE Trans. Neural Netw. Learn. Syst..

[11] Lim H.W., Tanjung S.Y., Iwan I., Yahya B.N., Lee S.L. (2025). FedEach: Federated Learning with Evaluator-Based Incentive Mechanism for Human Activity Recognition. Sensors.

[12] Xu J., Yao H., Zhang R., Mai T., Huang S., Xiong Z., Niyato D. (2024). Semantic-aware UAV swarm coordination in the metaverse: A reputation-based incentive mechanism. IEEE Trans. Mob. Comput..

[13] Lotfi I., Qaraqe M., Ghrayeb A., Niyato D. (2025). Vmguard: Reputation-based incentive mechanism for poisoning attack detection in vehicular metaverse. IEEE Trans. Veh. Technol..

[14] Deng L., Wang R., Liao Y., Xu R., Wang C. (2025). The reputation-based reward mechanism promotes the evolution of fairness. Appl. Math. Comput..

[15] Almeida L., Teixeira R., Baldoni G., Antunes M., Aguiar R.L. (2025). Federated Learning for a Dynamic Edge: A Modular and Resilient Approach. Sensors.

[16] Kang J., Yu R., Huang X., Wu M., Maharjan S., Xie S., Zhang Y. (2018). Blockchain for secure and efficient data sharing in vehicular edge computing and networks. IEEE Internet Things J..

[17] Chen Y., Zhang Y., Wang S., Wang F., Li Y., Jiang Y., Chen L., Guo B. (2022). Dim-ds: Dynamic incentive model for data sharing in federated learning based on smart contracts and evolutionary game theory. IEEE Internet Things J..

[18] Wang G., Dang C.X., Zhou Z. (2019). Measure contribution of participants in federated learning. Proceedings of the 2019 IEEE International Conference on Big Data (Big Data).

[19] Zhu Y., Liu Z., Wang P., Du C. (2023). A dynamic incentive and reputation mechanism for energy-efficient federated learning in 6g. Digit. Commun. Netw..

[20] Zhang C., Shen T., Bai F. (2022). Toward secure data sharing for the IoT devices with limited resources: A smart contract-based quality-driven incentive mechanism. IEEE Internet Things J..

[21] Liu S., Liu Z., Chen B., Pan X. (2024). Construction and application of online learning resource incentive mechanism driven by smart contract. IEEE Access.

[22] Yu Z., Chang Z., Wang L., Min G. (2024). Contract-Based Incentive Design for Resource Allocation in Edge Computing-Based Blockchain. IEEE Trans. Netw. Sci. Eng..

[23] Wang Z., Zhang W., Wang R., Liu Y., Xu C., Yu C. (2023). Smart contract based DDoS attack traceability audit mechanism in intelligent IoT. China Commun..

[24] Yue K., Zhang Y., Chen Y., Li Y., Zhao L., Rong C., Chen L. (2021). A survey of decentralizing applications via blockchain: The 5G and beyond perspective. IEEE Commun. Surv. Tutor..

[25] Wangni J., Wang J., Liu J., Zhang T. (2018). Gradient sparsification for communication-efficient distributed optimization. Adv. Neural Inf. Process. Syst..

[26] Jiao Y., Wang P., Niyato D., Lin B., Kim D.I. (2020). Toward an automated auction framework for wireless federated learning services market. IEEE Trans. Mob. Comput..

[27] Li Y., Li F., Yang S., Wu Y., Chen H., Sharif K., Wang Y. (2019). MP-coopetition: Competitive and cooperative mechanism for multiple platforms in mobile crowd sensing. IEEE Trans. Serv. Comput..

[28] Tang M., Wong V.W. (2021). An incentive mechanism for cross-silo federated learning: A public goods perspective. Proceedings of the IEEE INFOCOM 2021—IEEE Conference on Computer Communications.

[29] Rothchild D., Panda A., Ullah E., Ivkin N., Stoica I., Braverman V., Gonzalez J., Arora R. Fetchsgd: Communication-efficient federated learning with sketching. Proceedings of the International Conference on Machine Learning.

[30] Li L., Shi D., Hou R., Li H., Pan M., Han Z. (2021). To talk or to work: Flexible communication compression for energy efficient federated learning over heterogeneous mobile edge devices. Proceedings of the IEEE INFOCOM 2021—IEEE Conference on Computer Communications.

[31] Alistarh D., Hoefler T., Johansson M., Konstantinov N., Khirirat S., Renggli C. (2018). The convergence of sparsified gradient methods. Adv. Neural Inf. Process. Syst..

[32] Perazzone J., Wang S., Ji M., Chan K.S. (2022). Communication-efficient device scheduling for federated learning using stochastic optimization. Proceedings of the IEEE INFOCOM 2022—IEEE Conference on Computer Communications.

[33] Krizhevsky A., Hinton G. (2009). Learning Multiple Layers of Features from Tiny Images.

[34] Netzer Y., Wang T., Coates A., Bissacco A., Wu B., Ng A.Y. Reading digits in natural images with unsupervised feature learning. Proceedings of the NIPS Workshop on Deep Learning and Unsupervised Feature Learning.

[35] Xiao H., Rasul K., Vollgraf R. (2017). Fashion-mnist: A novel image dataset for benchmarking machine learning algorithms. arXiv.

[36] Zhan Y., Zhang J. (2020). An incentive mechanism design for efficient edge learning by deep reinforcement learning approach. Proceedings of the IEEE INFOCOM 2020—IEEE Conference on Computer Communications.

[37] Ding N., Fang Z., Huang J. (2020). Optimal contract design for efficient federated learning with multi-dimensional private information. IEEE J. Sel. Areas Commun..

[38] Khan L.U., Pandey S.R., Tran N.H., Saad W., Han Z., Nguyen M.N., Hong C.S. (2020). Federated learning for edge networks: Resource optimization and incentive mechanism. IEEE Commun. Mag..

